# Facial fold and crease development: A new morphological approach and classification

**DOI:** 10.1002/ca.23355

**Published:** 2019-03-07

**Authors:** Tudor Sandulescu, Marie Franzmann, Julia Jast, Tania Blaurock‐Sandulescu, Leoni Spilker, Caroline Klein, Ella A. Naumova, Wolfgang H. Arnold

**Affiliations:** ^1^ Department of Biological and Material Sciences in Dentistry School of Dentistry, Faculty of Health, Witten/Herdecke University Witten Germany

**Keywords:** facial fold, facial creases, wrinkles, superficial musculoaponeurotic system, facial fold classification

## Abstract

Facial folds and creases are established descriptive anatomical terms for structures of which the morphological characteristics and origins are not clearly defined. The aim of this study was to perform a morphological investigation of the nasolabial fold (NLF), mandibular fold (MF), deep transverse forehead (DTFC), infraorbital fold (IOF) and upper eyelid fold (UEF), correlating their phenotypes to differences in the superficial musculoaponeurotic system (SMAS), noting morphological differences and similarities. Full‐graft tissue blocks of skin, subcutaneous tissue, and mimic muscles collected postmortem were studied histologically. Serial histological sections were stained with Azan. Location‐ and composition‐specific morphological differences were determined. Histological serial section digitalization and three‐dimensional reconstruction of the tissue blocks were performed. Three different types of SMAS architecture were identified. Type I SMAS consisted of parallel‐aligned fibrous septa connecting the mimic muscles to the skin that covered the cheek, infraorbital and supraorbital, and forehead areas. Type II SMAS morphology appeared as a condensed Type I SMAS architecture with stronger fibrous septa and smaller fatty tissue compartments covering the lower and upper lip areas. Type III SMAS consisted of loose connective tissue covering the lower and upper eyelid regions. NLF, MF, IOF, and UEF are habitual primary folds induced by morphological changes in the underlying SMAS architecture. The secondary, accidental creases (DTFC) are cutaneous depressions derived from interacting dermal‐skeletal‐muscular changes without SMAS structure changes. The upper and lower eyelid wrinkles were tertiary, age‐related undulating skin redundancy formations. Clin. Anat. 32:573–584, 2019. © 2019 The Authors. *Clinical Anatomy* published by Wiley Periodicals, Inc. on behalf of American Association of Clinical Anatomists.

## INTRODUCTION

Facial surface topography has important meaning for all clinicians, medical students, and (increasingly) medical and forensic artists, anthropologists, and even lawyers (Dunn and Harrison 1997). Because Latin terminology has been avoided in clinical practice, and because of the International Anatomical Nomenclature Committee agreement regarding translation from the vernacular, uniform nomenclature has been lost (Dunn and Harrison 1997; Fabry et al. 2006). Unfortunately, there are various interchangeable terms for wrin, including “wrinkle,” “crease,” “furrow,” “line,” and “fold,” describing the same cutaneous formations (Mallouris et al. 2012; Hadi and Wilkinson 2017). Recent studies have demonstrated that the superficial musculoaponeurotic system (SMAS) defined a subcutaneous spreading musculoaponeurotic‐adipose layer covering the face with regional morphological differences probably related to nasolabial fold (NLF) development (Sandulescu et al. 2018a, 2018b, 2018c). Despite these histomorphological findings, the lack of uniform terminology has caused three different nomenclatures, “naso‐labial crease,” “naso‐labial groove,” and “naso‐labial fold,” to be used for the area marking the border between the cheek and the perioral area (Dunn and Harrison 1997; Hadi and Wilkinson 2017; Sandulescu et al. 2018a, 2018b, 2018c).

Epidermal thinning, loss of skin elasticity, fat compartment atrophy coupled with muscle pull and facial bone volume loss result in facial wrinkling and the formation of dynamic lines, NLFs, jowls, crow's feet and the sagging appearance of aged facial skin (Shaw Jr. et al. 2011; Gierloff et al. 2012a; Cotofana et al. 2016). Age‐related skin changes have been described with emphasis on changes in the subcutaneous fat compartments not yet including the SMAS architecture (Contet‐Audonneau et al. 1999; Mendelson et al. 2008; Gierloff et al. 2012b; Cotofana et al. 2016). Wrinkles define age‐related cutaneous changes associated with loss of skin elasticity, epidermal thinning, lowering of cell division in the stratum germinativum and flattening of the epidermal–dermal interface (Contet‐Audonneau et al. 1999; Akazaki et al. 2002; Luebberding et al. 2014; Kruglikov et al. 2016; Hadi and Wilkinson 2017). In the literature, the terms “crease” and “fold” refer to the same anatomical structures, describing fixed and permanent cutaneous visible anatomical landmarks characterized by skin attachment to the underlying tissue (Mallouris et al. 2012). Unfortunately, the terms “crease” and “fold” do not distinguish different anatomical structures (Dunn and Harrison 1997).

Facial crease morphology, biomechanical properties, and wrinkle severity all have been categorized by histological and various computer‐assisted optical methods, and clinical practitioners have attempted to use these characteristics to estimate age (Ernster et al. 1995; Takema et al. 1997; Contet‐Audonneau et al. 1999; Lemperle et al. 2001; Nouveau‐Richard et al. 2005; Fujimura et al. 2007; Tsukahara et al. 2007; Paes et al. 2009). Nevertheless, the lack of set nomenclature and the inconsistent application of the various terms leads to misinterpretation (Dunn and Harrison 1997). Therefore, as with SMAS description, a standardized facial crease nomenclature is necessary for effective scientific communications among clinicians, the scholarly community and researchers (Ghassemi et al. 2003; Hadi and Wilkinson 2017; Sandulescu et al. 2018a, 2018b, 2018c).

The aim of this study was to conduct a morphological investigation of facial folds and their relationships to the bordering SMAS architecture to establish a morphologically based definition and nomenclature of various fold phenotypes. The hypothesis was that SMAS architectural changes lead to facial fold development.

## METHODS

**Figure 1:**
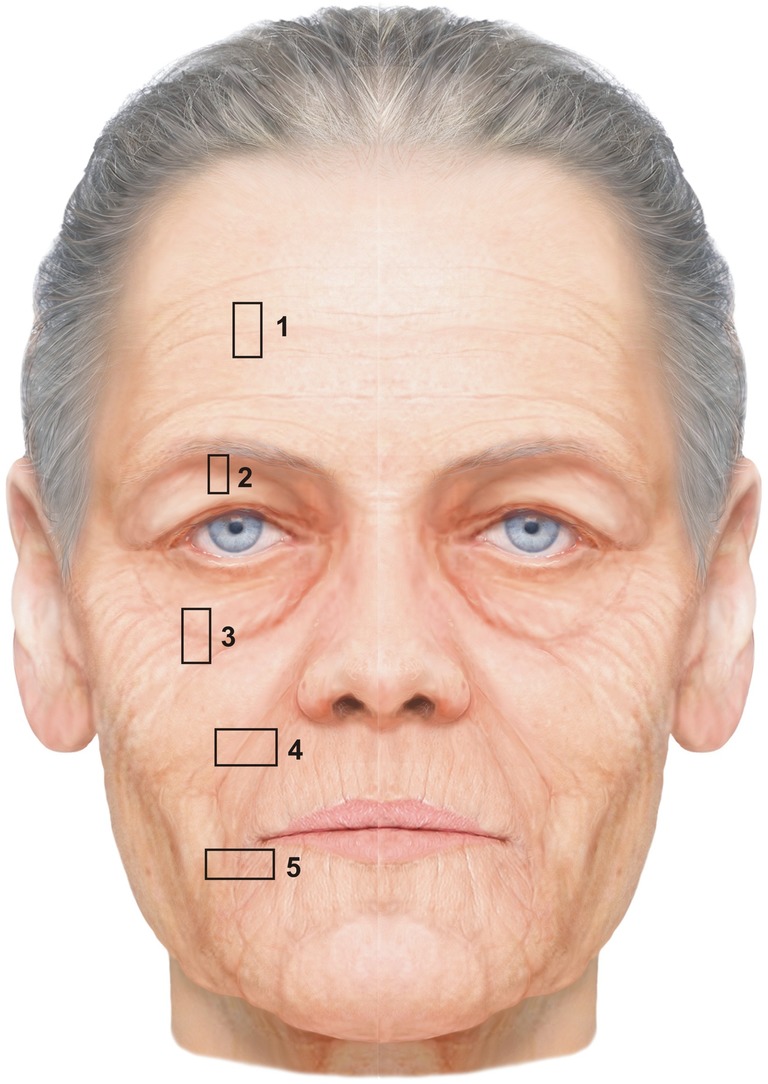
Schematic overview of the tissue block collection areas; 1‐DTFC; 2‐UEF; 3‐IOF; 4‐NLF; 5‐MF (modified from Radlanski and Wesker 2012). [Color figure can be viewed at http://wileyonlinelibrary.com]

Full‐graft tissue blocks of the skin, SMAS and mimic muscles of the NLF, infraorbital fold (IOF), upper eyelid fold (UEF), mandibular fold (MF), and deep transverse forehead crease (DTFC) region were collected postmortem from seven (three male and four female) donor bodies fixed in 4.5% formaldehyde (Fig. 1). The female and male donor bodies had average ages at death of 75.5 and 67.6 years, respectively. They were provided by the Department of Anatomy II, Friedrich‐Alexander‐Universität Erlangen‐Nürnberg, and were official testamentary donations of volunteers to the Department for the anatomy course for medical and dental students and for medical research purposes. The study was carried out according the regulations of the WMA Declaration of Helsinki in its present (2013) form. The donor sites showed no visible scars or tissue damage, and the medical histories revealed no surgical interventions or radiation to the head and neck area.

### Definition of Nomenclature Used

In the present manuscript, the following nomenclature was used:

- The term “fold” described cutaneous depressions related to SMAS morphological changes.
- The term “crease” described cutaneous depressions without SMAS morphological changes.
- The term “wrinkle” described age‐related undulating cutaneous relief formations consisting of redundant skin excess.

### Histological Analysis

After fixation in 4.5% formaldehyde, 1 cm × 2 cm × 1 cm tissue blocks containing skin, SMAS and mimic muscles were dissected and embedded in paraffin. Serial histological sections in the vertical plane of the NLF, IOF, UEF, MF, and DTF were cut to a thickness of 5 μm. Every section was collected and every 10th section was stained with Azan. Photomicrographs of the sections were taken with a Nikon D7000 camera (Nikon, Tokyo, Japan) with a resolution of 12 megapixels. The sections were also observed with a Leitz DMRB microscope (Leica, Wetzlar, Germany) and additional micrographs were taken.

### 3D Reconstruction

The photographs of the histological sections were consecutively imported into AutoCAD 2017 (Autodesk, Munich, Germany) and superimposed according to the best fit method. Skin, SMAS morphological structures (connective tissue fibers or fat compartments) and mimic muscles were two‐dimensionally digitized in separate layers. A 3D meshwork wire frame image was created for each structure. The 3D reconstruction and rendering were performed using 3ds Max 2017 (Autodesk, Munich, Germany). The 3D wire‐frame meshes were imported into 3ds Max, rendered into the models and visualized from various angles. AutoCAD and 3ds Max software was used to achieve fading out (freezing) and fading in of each digitized layer, so that individual interactions between different tissues without the interference of border layers could be analyzed. By digitally freezing or thawing single structures (electronic dissection) (Machin et al. 1996), the three‐dimensional architecture of SMAS structures and their relationships to the mimic musculature and the skin could be demonstrated. The three‐reconstructed figures had similar volumes to the tissue blocks used for histological analysis.

## RESULTS

### Macroscopy

Macroscopically, in all specimens, the NLF, MF, DTFC, IOF, and UEF were deep skin depressions. The NLF and MF marked the transition between the cheek and the upper lip and lower lip regions, respectively, while the IOF and UEF bordered the lower and upper eyelids from the infraorbital and supraorbital regions, respectively. The DTFC was identified as a prominent cutaneous depression horizontally covering the forehead area. Several flat parallel‐aligned skin depressions bordered the DTFC, MF, IOF, and UEF. The NLF had a straight structure in six donor bodies and a convex structure in one.

### Microscopy

#### Nasolabial fold

**Figure 2:**
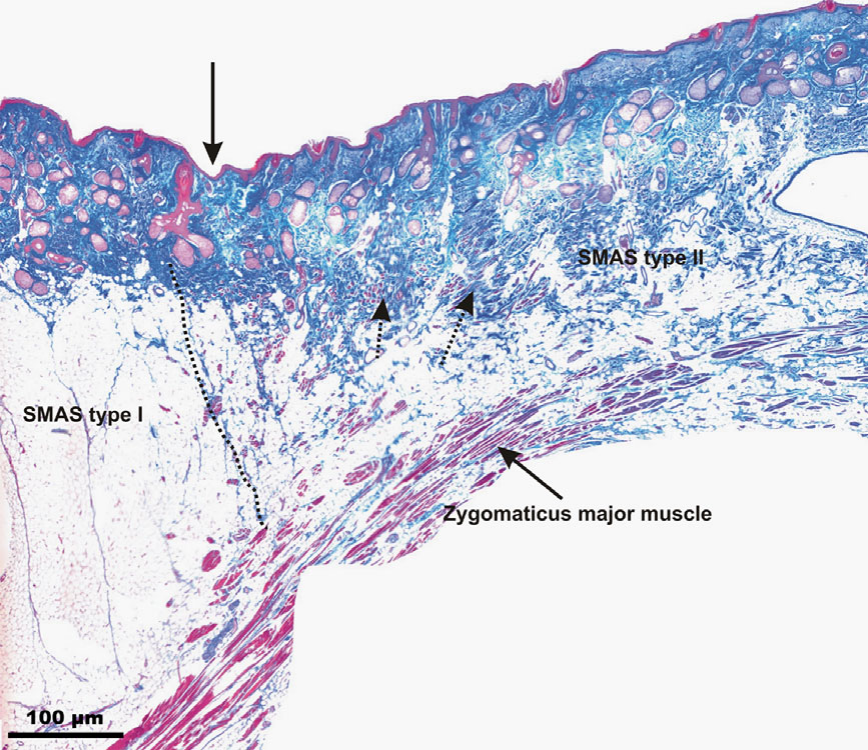
Microphotographic overview of the different SMAS morphologies in the NLF area. Type I SMAS covered the cheek region, lateral to the NLF, and Type II SMAS connected the zygomaticus major muscle to the skin medial to the NLF. Arrow marks the NLF; dotted arrows mark the various muscle cells in the Type II SMAS fibrous connective tissue fibers inserting into the dermis. The dotted line marks the border between SMAS Type I and SMAS Type II. [Color figure can be viewed at http://wileyonlinelibrary.com]

Microscopically, the NLF appeared as a deep cutaneous depression (Fig. 2). Lateral to it, the subcutaneous space was composed of parallel‐aligned fibrous connective tissue fibers vertical to the dermis forming fibrous septa connecting the mimic muscles to the skin (SMAS Type I). In the upper lip region, medial to the NLF, the fibrous septa condensed, building short strong connections between the zygomaticus major muscle and the skin (SMAS Type II). The spaces between the fibrous septa were filled with fat tissue. SMAS Type II morphology showed various muscular cells extending into the fibrous septa inserting directly into the dermis.

#### Mandibular fold

**Figure 3:**
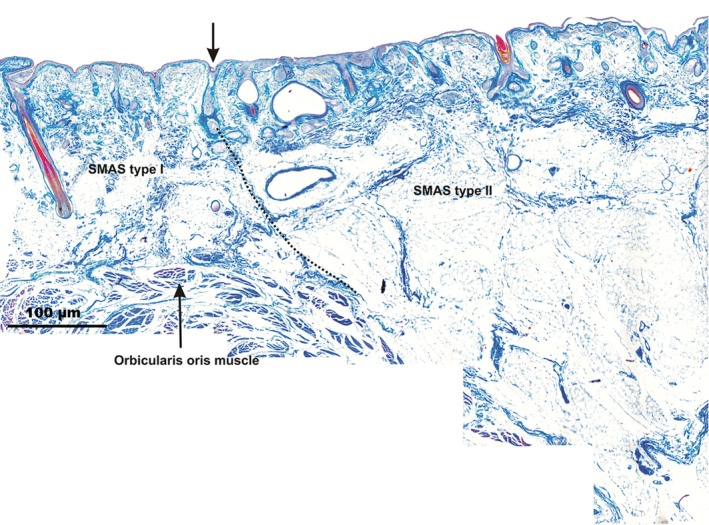
Microphotographic overview of the MF bordering Type I SMAS on the cheek side and Type II SMAS in the lower lip area; arrow marks the MF. On the lower lip side, SMAS fibrous fibers connect the orbicularis oris muscle to the skin. The dotted line marks the border between SMAS Type I and SMAS Type II. [Color figure can be viewed at http://wileyonlinelibrary.com]

As with the NLF, SMAS Type I architecture lateral to the MF in the cheek region changed its morphology to Type II SMAS on the lower lip side, medial to the MF (Fig. 3).

#### Infraorbital fold

**Figure 4:**
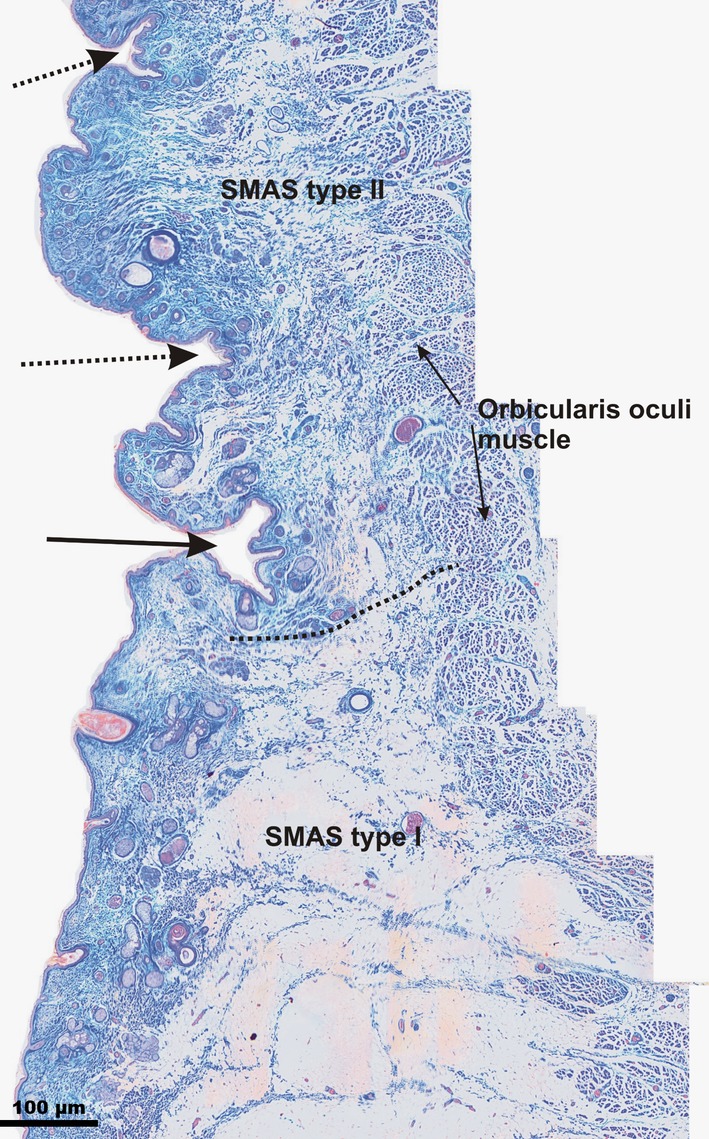
Microphotographic overview of the Type I and III SMAS bordering the IOF connecting the orbicularis oculi muscle to the skin. Type I SMAS covered the infraorbital area caudal to the IOF. Type III SMAS covered the lower eyelid area cranial to the IOF. Arrow marks the IOF; dotted arrows mark the cutaneous lower eyelid wrinkles. The dotted line marks the border between SMAS Type I and SMAS Type III. [Color figure can be viewed at http://wileyonlinelibrary.com]

The IOF cutaneous phenotype was a deep skin depression aligned along the infraorbital rim caudal to the infraorbital and cranial to the cheek area. Microscopically, the IOF was the most prominent cutaneous depression in the infraorbital region, strictly distinguishable from the surrounding wrinkles (Fig. 4). Caudal to it, in the infraorbital area, SMAS Type I showed similar morphological architecture to the subcutaneous tissue bridging the space between the orbicularis oculi muscle and the skin. In the lower eyelid region, cranial to the IOF, SMAS architecture changed, consisting of fat‐free loose connective tissue fibers connecting the orbicularis oculi muscle to the skin (SMAS Type III).

#### Upper eyelid fold

**Figure 5:**
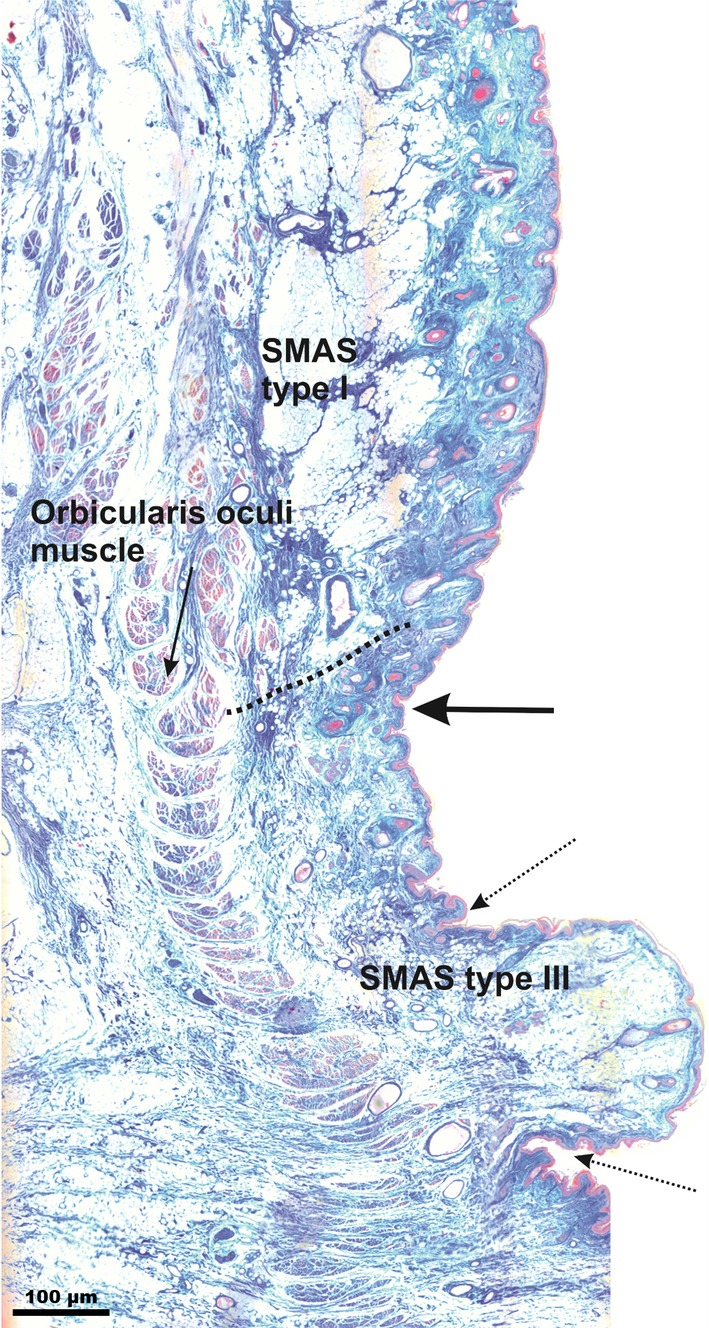
Microphotographic overview of the Type I and III SMAS bordering the UEF connecting the orbicularis oculi muscle to the skin. The dotted line marks the border between SMAS Type I and SMAS Type III. Type I SMAS covered the supraorbital area. Type III SMAS covered the upper eyelid area. Arrow marks the UEF; dotted arrows mark the cutaneous upper eyelid wrinkles. Between the wrinkles, cutaneous relief changed, appearing as an involutional effect with skin redundancy. [Color figure can be viewed at http://wileyonlinelibrary.com]

In all specimens, the UEF cutaneous formation could not be strictly distinguished from the neighboring wrinkles in the upper eyelid region (Fig. 5). Like the IOF, the UEF bordered Type I SMAS in the supraorbital area and Type III SMAS in the upper eyelid region. The bordering cutaneous wrinkles showed no morphological changes in the subcutaneous tissue. The bordering wrinkles consisted of involutional cutaneous formations with redundant skin excess.

#### Deep transverse forehead

**Figure 6:**
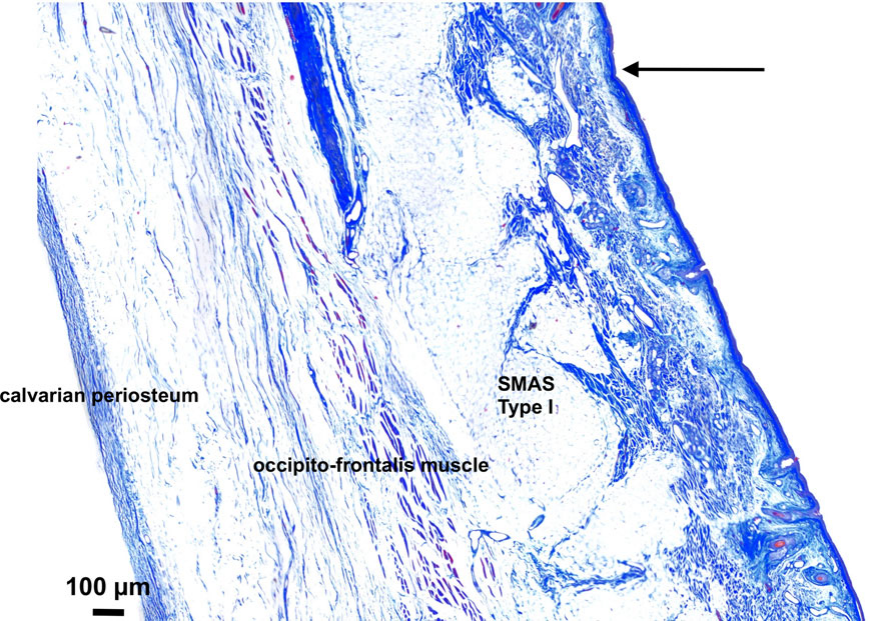
Microphotographic overview of the area bordering the DTFC. Arrow marks the macroscopically identified DTFC. Type I SMAS connected the occipitofrontalis muscle to the skin with no direct connection to the submuscular space or the underlying calvarial periosteum. [Color figure can be viewed at http://wileyonlinelibrary.com]

Microscopically, the DTFC was a flat cutaneous depression. Subcutaneous large fat compartments bolstered the spaces between the fibrous septa, connecting the occipitofrontalis muscle to the skin (Fig. 6). There was no connection between the forehead Type I SMAS fibrous septa and the underlying calvarial periosteum. The submuscular space was filled with loose connective tissue. The area underlying the DTFC showed no architectural changes in SMAS.

In view of the above findings, three adult SMAS types were distinguished:

#### Type I SMAS

**Figure 7:**
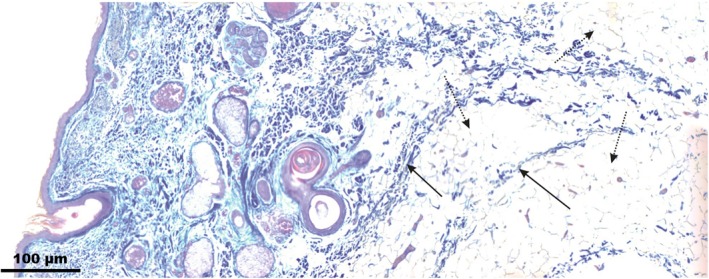
Microphotographic overview of Type I SMAS of the infraorbital region. Arrows mark the fibrous septa; dotted arrows mark the interseptal fat compartments. [Color figure can be viewed at http://wileyonlinelibrary.com]

Type I SMAS architecture consisted of parallel aligned fibrous septa connecting the mimic muscles to the skin. The interfibrotic spaces were bolstered with fat tissue (Fig. 7). Type I SMAS covered the area lateral to the NLF, the infraorbital, the supraorbital, and the forehead areas.

#### Type II SMAS

**Figure 8:**
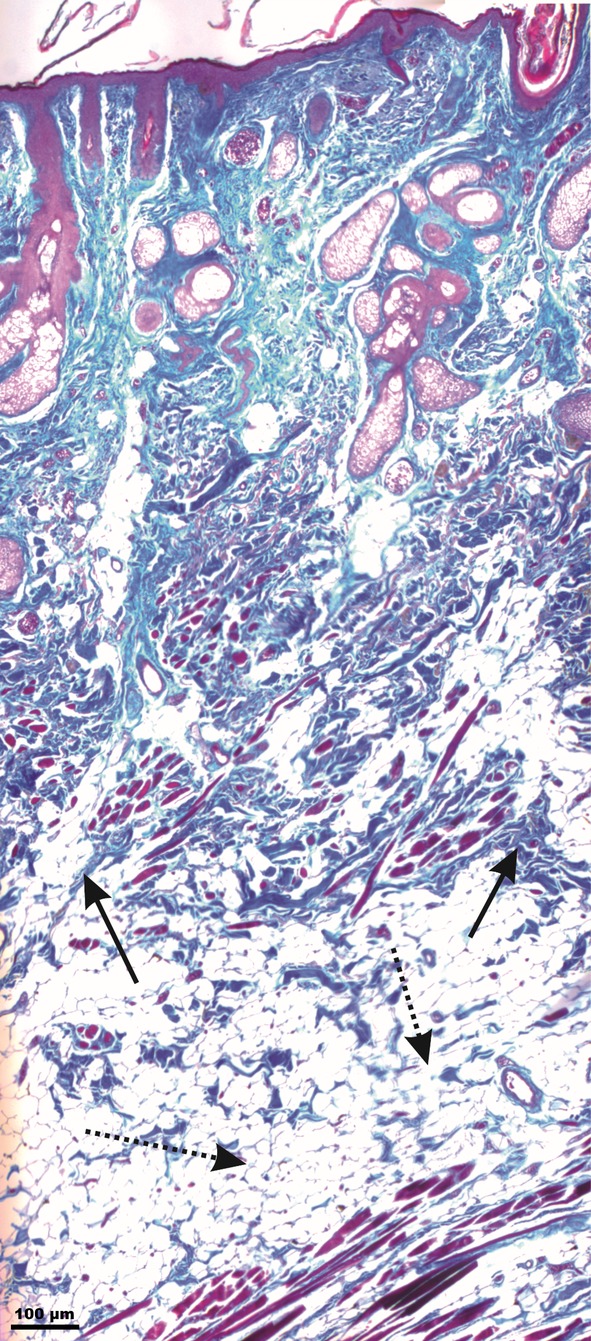
Microphotographic overview of Type II SMAS medial to the NLF. Arrows mark the fibrous septa with isolated muscle cells; stars mark the interseptal fat compartments. [Color figure can be viewed at http://wileyonlinelibrary.com]

In the upper and lower lip region, Type II SMAS was characteristic. Its morphology was similar to a condensation of Type I SMAS with short strong fibrous septa and smaller fat tissue compartments (Fig. 8).

#### Type III SMAS

**Figure 9:**
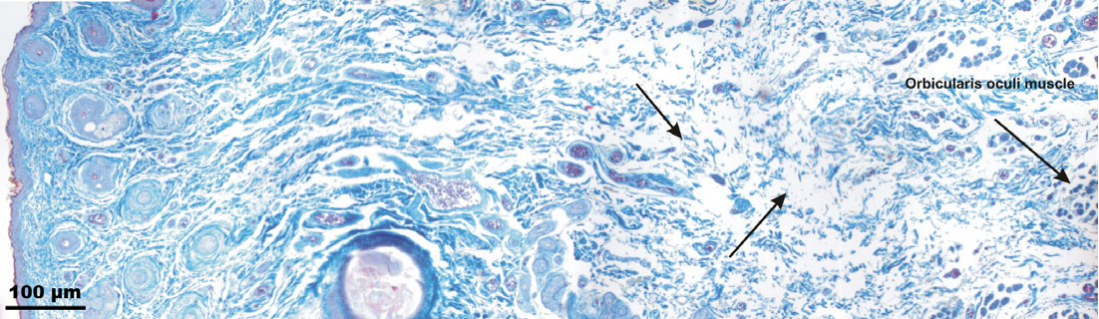
Microphotographic overview of Type III SMAS in the lower eyelid region. Arrows mark the loose connective tissue between the orbicularis oculi muscle and the skin. [Color figure can be viewed at http://wileyonlinelibrary.com]

Type III SMAS consisted of loose connective tissue and predominated in the lower and upper eyelid regions connecting the orbicularis oculi muscle directly to the skin (Fig. 9). Unlike Type I and Type II SMAS, no fat tissue was found in Type III SMAS.

### Digitalization, 2D Visualization, and 3D Reconstruction

**Figure 10:**
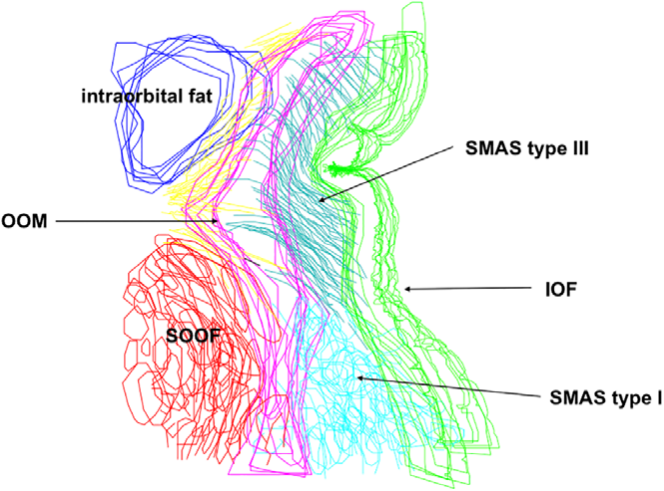
2D digitalization of the IOF bordering infraorbital Type I SMAS and lower eyelid Type III SMAS. Blue, intraorbital fat; Red, suborbicularis oculi fat (SOOF); OOM, orbicularis oculi muscle; Green, skin; Yellow, preseptal connective tissue. [Color figure can be viewed at http://wileyonlinelibrary.com]

**Figure 11:**
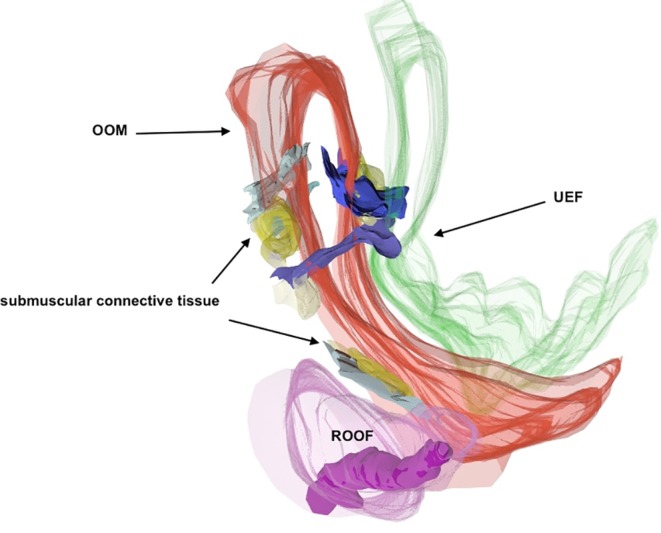
3D reconstruction of the UEF. The supraorbital SMAS Type I showed fibrous septa anchored in the OOM. Between the retro‐orbicularis oculi fat (ROOF) and the OOM was a gliding cushion of connective tissue. Green, skin; Red, OOM. [Color figure can be viewed at http://wileyonlinelibrary.com]

**Figure 12:**
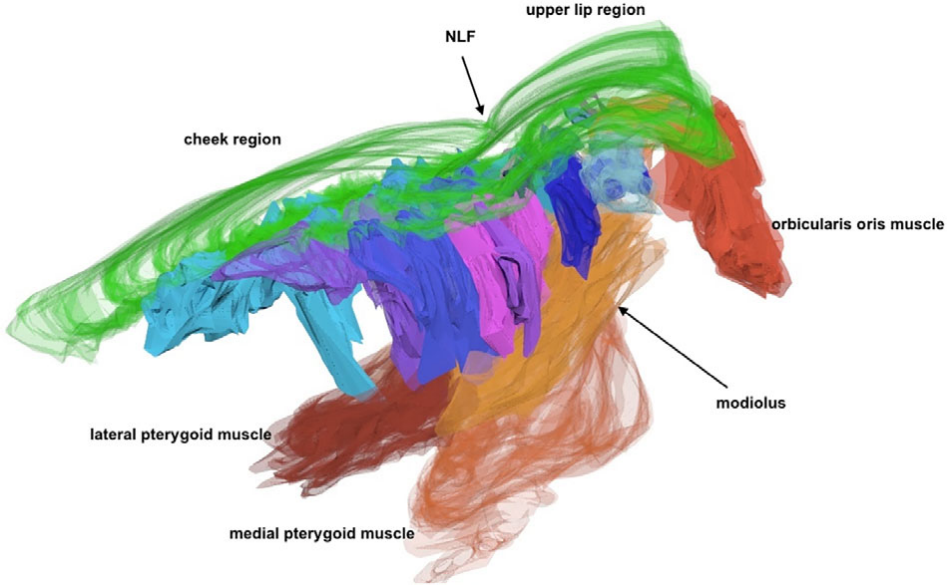
3D reconstruction of the NLF bordering cheek Type I SMAS and upper lip Type II SMAS. SMAS fibrous septa (blue tones) built a 3D meshwork connecting the mimic musculature to the skin. Fibrous septa (blue tones) built compartments bolstered by fatty tissue. Green, skin. [Color figure can be viewed at http://wileyonlinelibrary.com]

Fold 2D visualization and 3D reconstructions showed that the NLF, MF, UEF, and IOF lie between two different SMAS architectures. The cutaneous formation of these folds marked the border between the different SMAS morphology types. The UEF and IOF bordered supraorbital and infraorbital Type I SMAS and Type III SMAS of the eyelid regions, respectively (Figs. 10 and 11). The 3D reconstruction of the NLF showed that the SMAS fibrous tissue fibers were consecutively arranged, forming fibrous septa, and bordering Type I and Type II SMAS (Fig. 12). SMAS fibrous septa formed spaces bolstered with univacuolar fat tissue. Type I and Type II SMAS fat compartments were aligned parallel to the NLF. As with the NLF, SMAS morphology and septal arrangement could be demonstrated for the SMAS architecture around the MF.

**Figure 13:**
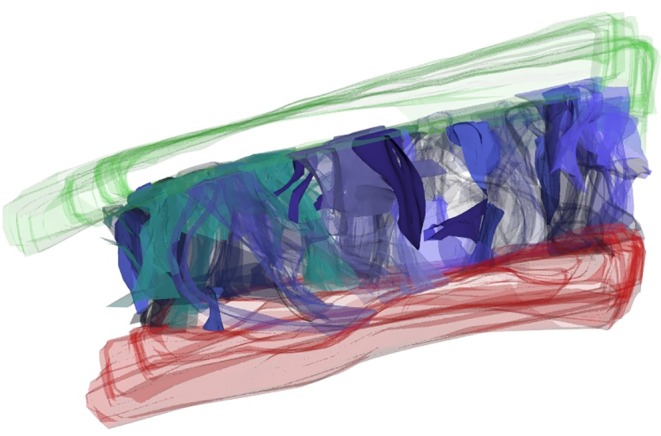
3D reconstruction of the forehead SMAS (Type I) showing vertical fibrous septa (blue tones) connecting the occipitofrontalis muscle (red) to the skin (green). The interseptal spaces were bolstered by fatty tissue. [Color figure can be viewed at http://wileyonlinelibrary.com]

The 3D reconstruction of the DTFC showed no changes in the underlying SMAS architecture. Frontal SMAS morphology was similar to cheek Type I SMAS architecture (Fig. 13).

## DISCUSSION

This study revealed new details about the morphological architecture of facial folds and creases. The method used combined 2D histological morphological analysis with 3D histological reconstruction. This allowed the morphological interaction between the mimic muscles, the SMAS and the skin to be analyzed histologically in 2D and 3D without tissue destruction during anatomical dissection. Virtual electronic dissection (Machin et al. 1996) revealed the interaction between the SMAS fibrous septa, mimic muscles, and the skin. In contrast to macroscopic dissection, this noninvasive virtual dissection helped to elucidate the 3D structural morphological interactions and architectural constitution without destroying the layer connections by surgical separation.

The results demonstrated that SMAS morphology is closely linked to facial fold formation. The development of facial folds, creases, and wrinkles is multifactorial and is assumed to be associated with biological aging, which involves involutionary processes such as loss of skin elasticity and changes in the volumes of underlying fat compartments, leading to a chronological age‐related classification (Akazaki et al. 2002; Albert et al. 2007; Gierloff et al. 2012a, 2012b; Luebberding et al. 2014; Cotofana et al. 2016; Kruglikov et al. 2016; Hadi and Wilkinson 2017; Suwanchinda et al. 2018). In contrast to the common classifications, our findings showed that additional morphological aspects such as SMAS architecture and its regional differences need to be considered and classified.

### Facial Folds and Creases

#### Nasolabial fold

The NLF is macroscopically situated on the cheek region starting from the tip of the ala of the nose and ending at the corner of the mouth, marking the transition to the perioral area (Lemperle et al. 2001;Sandulescu et al. 2018a, 2018b, 2018c). As one of the most investigated facial folds, the NLF was mentioned in earlier studies under various labels such as “melolabial fold” (Bagal et al. 2007; Gassner et al. 2008) or “nasomandibular fold” (Robbins et al. 1995). Previous studies investigated the phenotype of the NLF, describing its shape and angle, which could be concave, straight, or convex (Rubin et al. 1989; Zufferey 1992; Pessa et al. 1998). Our most recent published study analyzed the three‐dimensional aspect of the SMAS bordering the NLF and concluded, similar to the results of the present study, that the NLF defined the transition area between two SMAS morphologies: Type I in the cheek area and Type II in the perioral area (Sandulescu et al. 2018a, 2018b, 2018c). Furthermore, in the present study, there were no morphological differences in SMAS architecture between the straight and convex NLF phenotypes as described by Pessa and colleagues (Pessa et al. 1998). The histological 3D reconstruction of the SMAS surrounding the NLF showed that the SMAS fibrous septa formed microscopic communicating superficial fat compartments that were aligned parallel to the NLF, possibly explaining earlier macroscopic descriptions of a middle fat compartment situated lateral to the NLF (Pilsl and Anderhuber 2010a, 2010b; Gierloff et al. 2012a, 2012b). The histological findings did not confirm the existence of a superficial macroscopic independently dissectible fat compartment as described by Gierloff 2012a, 2012b and Pilsl 2010a, 2010b, similar to the suborbicularis oculi fat (SOOF) (Pilsl and Anderhuber 2010a, 2010b; Gierloff et al. 2012a, 2012b; Sandulescu et al. 2018a). In conclusion, the somatic correlates for the development of the NLF are represented by the border between various SMAS architectures. According to the morphological findings, the term “nasolabial fold” should be established.

#### Mandibular fold

As with the NLF, the nomenclature of the MF includes various terms such as “marionette line(s)” (Dunn and Harrison 1997; Carruthers and Carruthers 2010), “jowls” (Reece and Rohrich 2008), “melomental folds” (Bagal et al. 2007), “labiomandibular fold” (Pessa et al. 1998), and “labiomandibular sulcus” (Suwanchinda et al. 2018). The MF arises from a multifactorial effect during the interaction between the submandibular and mandibular septum fat compartments, the retaining ligaments, and the overlying skin (Reece and Rohrich 2008; Gierloff et al. 2012b; Kruglikov et al. 2016; Suwanchinda et al. 2018). The macroscopic investigation by Suwanchinda et al. (2018) described, during dissection, loose connections between the skin and the fat compartment lateral to the labiomandibular sulcus and strong adhesion between the medial fat compartment and the skin (Suwanchinda et al. 2018). The histological finding in the present study supports the macroscopic observations of Suwanchinda et al. (2018). SMAS Type I and II morphological architectures medial and lateral to the MF explained the different macroscopic dissection properties. Furthermore, the histological results showed, as for the NLF, an architectural change in the SMAS morphology surrounding the MF. Therefore, we conclude that SMAS morphological changes should be considered and implemented into the developmental mechanism of the MF and, by analogy with the NLF, the label “mandibular fold” nomenclature should be established.

#### IOF and UEF

The IOF is situated at the border between the infraorbital and cheek areas following the lower margin of the infraorbital rim (Hadi and Wilkinson 2017). Similar notations for the IOF (Sandulescu et al. 2018a) have been proposed, such as “nasojugal groove” (Camp et al. 2011) or “nasojugal fold” (Shaw Jr. et al. 2011). The IOF was localized using cutaneous landmarks such as the mid‐pupillary line and the medial canthal line (Camp et al. 2011). The results of other studies did not define the IOF (Shaw Jr. et al. 2011). The UEF is formed by the subcutaneous insertion of the terminal fibers of the levator aponeurosis (Mallouris et al. 2012).

In this study, we recognized that the IOF, such as the NLF and MF, was bordered by two different SMAS morphological architectures. The IOF marked the border between the Type I SMAS of the cheek region and Type III SMAS of the lower eyelid area. Therefore, standardization of the nomenclature to “infraorbital fold” has been proposed. Similar SMAS morphological changes have been demonstrated for the UEF, so the cutaneous levator aponeurosis insertion is not the only reason for formation of the UEF. In summary, the IOF and UEF are determined by SMAS architectural changes that can be morphologically differentiated from the bordering lower and upper eyelid wrinkles.

#### Deep transverse forehead crease

Creases of the forehead cover the frontal bone and are almost horizontal in pattern (Hadi and Wilkinson 2017). Typical forehead crease nomenclature includes “horizontal forehead creases” (Albert et al. 2007), “forehead lines” (Carruthers and Carruthers 2010), or “horizontal forehead lines” (Lemperle et al. 2001). In the present study, the DTFC was macroscopically a prominent and deep horizontally aligned transverse skin depression over the forehead area located at various heights between the superior orbital rim and the hairline. Subcutaneous tissue analysis revealed no morphological SMAS architecture changes responsible for development of the DTFC. Therefore, we concluded that the DTFC was a cutaneous depression determined by interacting aging‐related dermal‐skeletal‐muscular changes (Albert et al. 2007) without SMAS architectural predilections. Therefore, “deep transverse forehead crease” was an appropriate nomenclature for all horizontally aligned transverse forehead skin depressions.

#### SMAS architecture analysis and classification

Analysis of the subcutaneous tissue demonstrated three different SMAS morphological types: Type I in the cheek and forehead region, Type II in the perioral region medial to the NLF and MF, and Type III covering the upper and lower eyelid regions caudal to the UEF and cranial to the IOF. The existence of Type I SMAS covering the perioral region and Type II SMAS covering the midfacial and the forehead areas was demonstrated in early studies by Ghassemi et al. (2003) and corroborated by our latest studies (Ghassemi et al. 2003; Sandulescu et al. 2018a, 2018b, 2018c). A previous study described Type III SMAS in the lower eyelid area cranial to the IOF (Sandulescu et al. 2018a). In the current study, a similar Type III SMAS morphology was demonstrated in the upper eyelid region caudal to the UEF. In conclusion, the periocular region was covered by a continuous SMAS tissue with two different morphologies. Type I SMAS parallel‐aligned fibrous septa connected the orbicularis oculi muscle to the skin caudal to the IOF and cranial to the UEF. As revealed in former studies, Type III SMAS consisting of fat‐free loose fibroelastic connective tissue connected the orbicularis oculi muscle to the skin (Sandulescu et al. 2018a). We assumed that the fibrous connections transfer muscle contraction to the skin level, deepening the UEF and the IOF as described in the literature (Mallouris et al. 2012). The cutaneous phenotypes of the periocular folds and wrinkles were macroscopically and microscopically similar and could not be differentiated by their prominence or depth. Microscopic examination of the underlying SMAS architecture helped identify the IOF and the UEF and their discrimination from the bordering upper and lower eyelid wrinkles.

In conclusion, the UEF and the IOF are constant habitual folds with morphological changes of SMAS tissue as predilection factors. The bordering upper and lower eyelid wrinkles were assumed to result from an aging‐related undulating skin involutional effect. Therefore, the upper and lower eyelid wrinkles were categorized as cutaneous redundancy formations.

Periorbital aging was described as a result of multiple changes in skin color and consistency, subcutaneous fat atrophy and changes in the underlying bony structures (Camp et al. 2011). The individual importance of each of these changes remains incompletely understood (Anastassov and St Hilaire 2006; Camp et al. 2011), although recent studies have demonstrated direct interactions among the periorbital submuscular fat compartments such as the SOOF, the orbicularis oculi muscle, the periorbital SMAS, and the skin (Sandulescu et al. 2018a). For clinical practice, it is assumed that folds and wrinkles can be treated with augmentative and ablative surgical procedures, respectively, while softening of the IOF involves subcutaneous manipulations respecting the various SMAS morphologies. As in the procedure described by Wang and Huang (2011) for softening the NLF, loosening the transition zone between types I and III SMAS of the IOF could induce a similar cutaneous effect leveling the deep fold (Wang and Huang 2011).

By analogy with the periocular findings, the perioral and the midfacial region lateral to the NLF showed similar SMAS morphological changes. Type I SMAS in the midfacial region and Type II SMAS in the perioral area bordered the NLF and the MF.

In conclusion, the NLF, MF, IOF, and UEF are habitual cutaneous depressions determined by structural differences between the bordering SMAS regions. This classification supports the observation of Mallouris et al. (2012), in which the NLF was visible in patients with muscular paralysis (Mallouris et al. 2012).

In contrast to the above findings, the DTFC could not be clearly identified in the histological sections, although it was demonstrated macroscopically in all donor bodies before the tissue blocks were harvested. Histological analysis of the SMAS architecture demonstrated similar Type I morphology of the fibrous tissue connecting the occipitofrontalis muscle to the skin. There were no changes in SMAS morphology along the tissue block. These results led to the conclusion that the DTFC was an accidental cutaneous depression without predisposing subcutaneous morphological SMAS changes, as demonstrated for the habitual UEF, IOF, NLF, and MF.

In conclusion, this study demonstrated that SMAS morphology determines the development of habitual folds. The NLF and MF marked the transitional area between Type I SMAS of the cheek region and the periorally located Type II SMAS. The IOF and UEF marked the change in subcutaneous SMAS architecture between Type I SMAS of the infraorbital and supraorbital regions and the lower and upper eyelid regions, respectively. The NLF, MF, IOF, and UEF proved to be habitual, with ubiquitous incidence and somatic cutaneous and subcutaneous correlates. Therefore, SMAS architectural changes are a conditio sine qua non for habitual facial fold development. Other than age‐related involutionally determined facial wrinkles, SMAS architectural changes represent habitual facial fold origins and predilections. Unlike a former analysis that described cutaneous relief, the results of this study present a new point of view regarding the development of facial folds by analyzing the subcutaneous SMAS architecture and its changes. The DTFC showed no SMAS morphological changes, so it was assumed that it was accidentally developed and determined by aging‐related involutional processes combined with interactions among the skin, occipitofrontalis muscle contraction, and the SMAS.

The habitual UEF and IOF correspond to facial landmarks described by Lambros (2007), according to whom they show no significant movement during the aging process (Lambros 2007). By analogy with Lambros's (2007) observation combined with our morphological results, we assumed that the NLF and MF have similar aging properties to the periorbital folds because of the similar underlying SMAS architectural changes.

The hypothesis of this study was that SMAS architectural changes lead to facial fold development. This hypothesis was confirmed for the NLF, MF, IOF, and UEF and was refuted for the DTFC.

#### Future analogical developmental hypothesis

Facial fold and crease formation is a fascinating and poorly understood developmental process similar to gyrification (White et al. 2010). We hypothetically proposed an analogy of facial folds and creases to cerebral sulci because of the similarity of morphological developmental stages (White et al. 2010). As with the cerebral sulci, facial folds and creases develop and become prominent during growth (White et al. 2010).

The classification used in the literature describes cutaneous primary creases as visible to the eye and forming polygons. Secondary creases divide those polygons into triangular areas that are subdivided by tertiary creases that extend deeply to the epidermis (Mallouris et al. 2012). The cerebral sulci are classified according to the cortical folding process as primary, secondary, and tertiary (Филимонов 1953; Филимонов 1955). Primary and secondary sulci were related to predisposition and genetic factors, and tertiary sulci were the result of aging‐related changes (Филимонов, 1953).

In our opinion, primary cutaneous formations that correspond to habitual facial folds (NLF, MF, UEF, and IOF) are closely related to morphological structural predispositions, such as the SMAS architecture and its changes. Secondary cutaneous formations are represented by accidental creases without subcutaneous architectural correlates. The tertiary facial cutaneous formations consisted of the age‐related skin redundancy formations represented by wrinkles.

Like facial folds, cerebral sulci are landmarks of which identification is mandatory for surgical interventions (Ribas 2010; Camp et al. 2011).

## CONCLUSIONS

In this study, the importance of SMAS morphological analysis for facial fold classification and categorization of the aging face has been demonstrated. A noninvasive histological 3D reconstruction has been presented, allowing virtual histological dissection to be performed and the morphological architecture of each single tissue and its interactions with the bordering structures to be understood. Furthermore, the results of the present study provided a ubiquitously applicable morphology dependent on customized facial fold and crease classifications.

Adult folds were categorized as habitual and accidental cutaneous formations. Habitual folds such as the NLF, MF, UEF, and IOF showed subcutaneous SMAS morphological changes. The accidental skin formations (DTFC) were cutaneous depressions without predictive SMAS structural changes. The upper and lower eyelid wrinkles were age‐related cutaneous redundancy formations.

The highlight of this study is the new facial fold classification: primary cutaneous formations (folds) are habitual cutaneous formations showing changes in the bordering SMAS architecture (NLF, MF, UEF, and IOF); secondary cutaneous formations (creases) are accidental, having no subcutaneous SMAS architectural changes (DTFC); and tertiary folds are cutaneous redundancy formations (upper and lower eyelid wrinkles).

We hope that this study will lead to an improved understanding of facial fold morphology and development.

## CONFLICT OF INTERESTS

The authors declare that they have no conflict of interests.

## AUTHOR CONTRIBUTIONS

TS wrote the manuscript. MF performed the UEF histological analysis and 3D reconstruction. JJ performed the DTF histological analysis and 3D reconstruction. TBS performed the IOF histological analysis and 3D reconstruction. LS performed the NLF histological analysis and 3D reconstruction. CK performed the MF histological analysis. EAN corrected the manuscript. WHA supervised the project, 3D rendering, and final manuscript approval.

## ETHICAL DECLARATION

The study was carried out according to the regulations of the WMA Declaration of Helsinki in its present (2013) form.
